# Bio-protection in oenology by *Metschnikowia pulcherrima*: from field results to scientific inquiry

**DOI:** 10.3389/fmicb.2023.1252973

**Published:** 2023-08-17

**Authors:** Maëlys Puyo, Scott Simonin, Benoit Bach, Géraldine Klein, Hervé Alexandre, Raphaëlle Tourdot-Maréchal

**Affiliations:** ^1^UMR Procédés Alimentaires et Microbiologiques, Institut Agro Dijon, Université de Bourgogne Franche-Comté, Équipe Vin Alimentation Micro-Organismes Stress (VAlMiS), Dijon, France; ^2^Changins, Viticulture and Enology, HES-SO University of Applied Sciences and Arts Western Switzerland, Nyon, Switzerland

**Keywords:** bio-protection, *Metschnikowia pulcherrima*, oenology, microbial interactions, non-*Saccharomyces*

## Abstract

Finding alternatives to the use of chemical inputs to preserve the sanitary and organoleptic quality of food and beverages is essential to meet public health requirements and consumer preferences. In oenology, numerous manufacturers already offer a diverse range of bio-protection yeasts to protect must against microbiological alterations and therefore limit or eliminate sulphites during winemaking. Bio-protection involves selecting non-*Saccharomyces* yeasts belonging to different genera and species to induce negative interactions with indigenous microorganisms, thereby limiting their development and their impact on the matrix. Although the effectiveness of bio-protection in the winemaking industry has been reported in numerous journals, the underlying mechanisms are not yet well understood.

The aim of this review is to examine the current state of the art of field trials and laboratory studies that demonstrate the effects of using yeasts for bio-protection, as well as the interaction mechanisms that may be responsible for these effects. It focuses on the yeast *Metschnikowia pulcherrima*, particularly recommended for the bio-protection of grape musts.

## 1. Introduction

Current concerns to reduce chemical pesticides, fungicides and bactericides in the agri-food industry have encouraged researchers and industries to develop new strategies over the last 20 years. One of them consists in adding microorganisms after harvest to control spoilage microorganisms: this is the biocontrol concept mainly used in agriculture for the protection of fruits and vegetables (Spadaro and Gullino, 2004; Droby et al., 2009; Sharma et al., 2009). Non-*Saccharomyces* (NS) yeasts have been given great attention due to their microbial antagonisms that biologically inhibit potential post-harvest moulds (Comitini et al., 2023). The development of this approach has more recently been extended to the field of oenology, as an alternative to the use of sulphites to protect musts, mainly from potential alteration by indigenous microbiota at the pre-fermentative step. This is the strategy of bio-protection (García et al., 2016), corresponding to the implementation of non-*Saccharomyces* (NS) yeasts as in biocontrol but in winemaking framework. However, the function of a strain used in biocontrol differs from that expected of a strain recommended for bio-protection. Indeed, a bioprotective strain in oenology has to be effective against specific microorganisms considered as potential spoilage agents (such as *Hanseniaspora* and *Brettanomyces* yeasts, and lactic acid bacteria) in specific environmental conditions linked to the first steps of transforming grapes into must (acidity of must, low temperature and levels of oxygen).

In winemaking, NS yeasts have long been considered as undesirable microorganisms, responsible for sluggish or incomplete fermentations and the production of unpleasant aromas (Bisson, 1999), thus leading to economic losses for winemakers. However, the focus on these yeasts which are largely predominant on grape berry and must at the beginning of alcoholic fermentation (Albertin et al., 2014; Garofalo et al., 2016; Mateus et al., 2020) has changed over the last few decades, and NS yeasts including *Metschnikowia pulcherrima* are now marketed in Active Dry Yeast form (ADY) (Roudil et al., 2020). *Metschnikowia pulcherrima* strains were first recommended in winemaking for their contribution to the aromatic development of wine through their enzymatic activities (β-D-glucosidase, cysteine β-lyase) (Charoenchai et al., 1997; Morata et al., 2019) and the production of a wide range of metabolites (esters, higher alcohols) resulting from alcoholic fermentation. An extensive bibliography highlighted the positive effects of *M. pulcherrima*, tested on different grape varieties (Sauvignon blanc, Chenin blanc, Muscat, Merlot) in sequential inoculation with *Saccharomyces cerevisiae*, on the organoleptic qualities of wines (Zohre and Erten, 2002; Jolly et al., 2003; Rodríguez et al., 2010; Comitini et al., 2011; Zott et al., 2011; Sadoudi et al., 2012; Morales et al., 2015; Zhang et al., 2020; Aplin et al., 2021; Lin et al., 2022). However, some strains have been described as producing off-flavour, such as ethyl acetate leading to unpleasant banana or glue flavours in wine (Cosme et al., 2018), underlining the importance of strain selection for oenological application. More recently, the use of *M. pulcherrima* for the bio-protection of grape must has been proposed to winemakers as an alternative to using sulphites. A new positive function of *M. pulcherrima* is now being highlighted: an antagonistic role against indigenous grape microbiota during the pre-fermentation phases without necessarily impacting on the final organoleptic properties of the wines.

This review presents the state of the art in the application and effectiveness of bio-protection by *M. pulcherrima* in cellar conditions. An investigation into the mechanisms potentially involved in the bioprotective effect of this yeast, such as the production of antimicrobial compounds and enzymatic activities is proposed. It also focuses on data relating to *M. pulcherrima*’s nutritional needs which could be implicated in its bioprotective effect through nutrient competition.

## 2. Bio-protection by *Metschnikowia pulcherrima* yeast: effectiveness in field trials

The strains available on the market have been phenotypically characterised under oenological conditions and meet the criteria imposed by the specifications allowing their use in winemaking (Roudil et al., 2020).

*Metschnikowia pulcherrima* strains are those most frequently tested for bio-protection, pure or associated with *Torulaspora delbrueckii* (Table 1). Not only do these yeasts have antagonistic effects on other indigenous yeast species, they also appear to be cold-resistant, making them particularly effective in the pre-fermentation phases conducted at low temperatures. Furthermore, Grazia et al. (2019) showed that *M. pulcherrima* could tolerate SO_2_ concentration ranging from 125 to 200 mg/L, which is higher than the concentration used in standard winemaking process.

**Table 1:** *Metschnikowia pulcherrima* yeasts marketed as NS yeast strains for bio-protection.

Products	Companies	Yeast species	Interest in winemaking (company sources)
PRIMAFLORA VB^®^ (>2017) and PRIMAFLORA VR^®^	AEB	*M. pulcherrima*	Microbiologically protects the musts. Contributes to the flavour complexity
ZYMAFLORE^®^ EGIDE	Laffort	*T. delbrueckii + M. pulcherrima*	Bio-protection and pre-fermentation control
ZYMAFLORE^®^ KHIO	Laffort	*M. pulcherrima*	Suitable for pre-fermentation phases at low temperatures
Excellence^®^ BIO-NATURE	Lamothe-Abiet	*M. pulcherrima*	Protects the must from indigenous microbiota
LEVEL^2^INITIA™	Lallemand	*M. pulcherrima*	Double action of oxygen: consumption and reduction of copper levels

The implementation protocols for bio-protectants containing *Metschnikowia* strains are consistent across different companies, with all products available in dry form. The rehydration protocol is similar to that used to prepare *S. cerevisiae*. Regardless of the strain used, seeding rates typically range from 5 g/qt on grapes to 3–5 g/hL on juice, resulting in values between 5.10^5^ and 10^6^ Colony Forming Unit (CFU)/mL. It is recommended to apply the product as early as possible, either in the bin or during vatting, or even by spraying during mechanical harvesting. In the case of white grapes, the product can be applied just after pressing.

**Figure 1:**
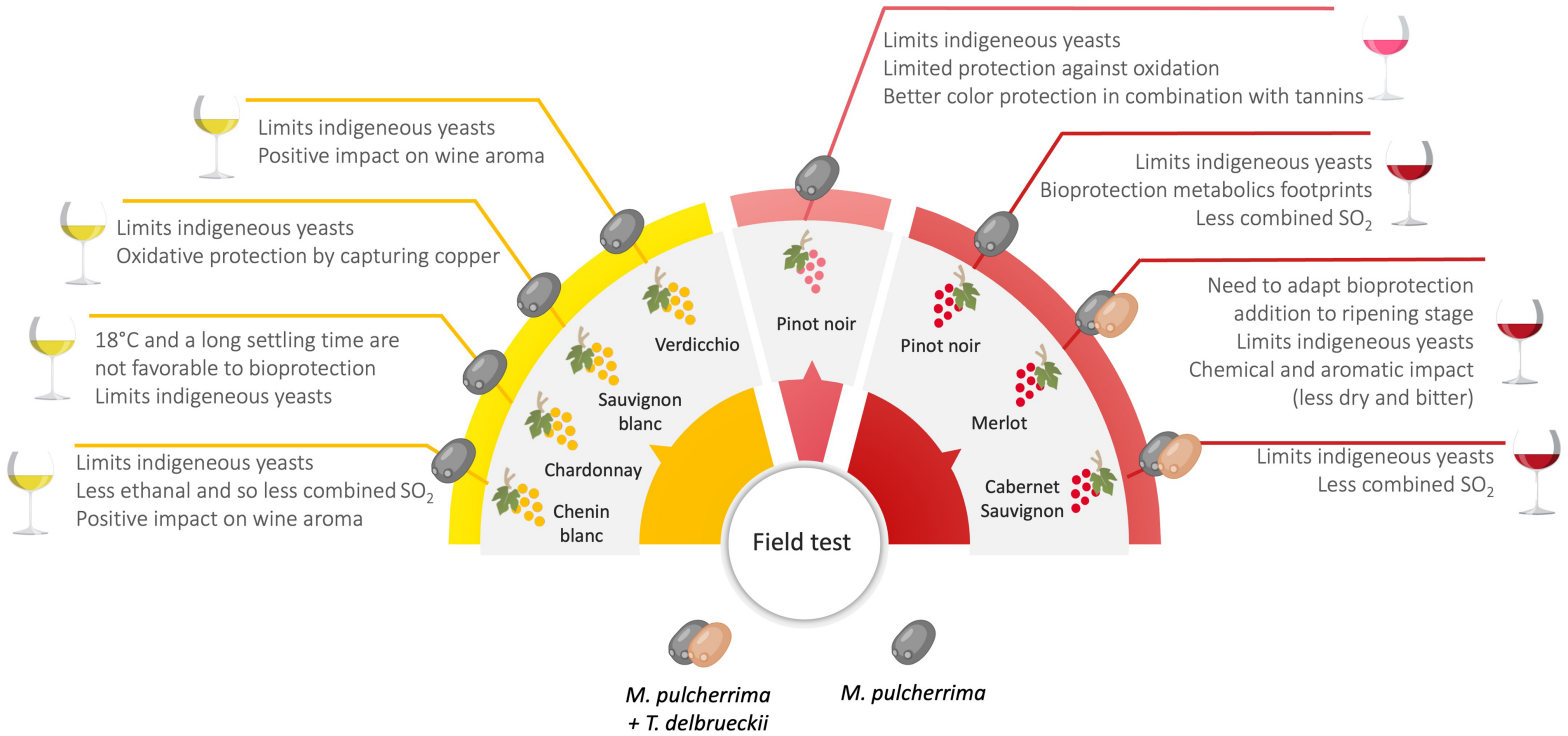
Main results on bio-protection obtained in field trials (From data published in journals for professionals and in Chacon-Rodriguez et al., 2020; Simonin et al., 2020, 2022; Windholtz et al., 2021a,b,c; Agarbati et al., 2023; Puyo et al., 2023).

Although many technical reports have underlined the positive results obtained by the bio-protection strategy, the first scientific evidence highlighting the efficiency of bio-protective strains have become available only recently. Figure 1 provides some examples of experiments that demonstrate the efficacy of bio-protection under cellar conditions.

In white winemaking, a *M. pulcherrima* strain was tested on Chardonnay must using three temperatures (7°C, 12°C and 18°C) and two settling times (36 and 72 h) in order to determine the optimal conditions for a bioprotective effect (Simonin et al., 2022). The results showed a considerable impact of temperature on the bioprotectant implantation. On must with a settling time of 72 h at 7°C, *M. pulcherrima* represented 99% of the total yeast concentration, with only 1% of *Hanseniaspora uvarum*, and the inhibition of *Brettanomyces bruxellensis* which became undetectable. The implantation of *M. pulcherrima* during a cold static clarification of a Verdicchio must at low temperature (10°C, 24 h) and its control on indigenous wild yeast populations were confirmed by the work of Agarbati et al. (2023). However, an increase in settling temperature to 18°C did not allow the implantation of the bio-protection strain and therefore the control of indigenous flora (Simonin et al., 2022). The impact of bio-protection on the organoleptic qualities of wines gave contradictory results, depending on the grape varieties tested. Tested on Chardonnay, the bio-protection of must did not have a significant influence on the chemical and sensory characteristics of wines (Simonin et al., 2022), whilst on Verdicchio must, the application of bio-protection induced a specific aromatic imprint in the wine due to volatile compounds and confirmed by sensorial analyses (Agarbati et al., 2023).

The bio-protection strategy was also used during red winemaking on different grape varieties. On Pinot noir, the addition of a *M. pulcherrima* strain during the cold pre-fermentative stage limited the growth of indigenous flora, like for white musts (Simonin et al., 2020). On Cabernet Sauvignon, the inoculation of a combination of the bioprotective strains *M. pulcherrima* and *T. delbrueckii* after grape vatting and maceration at 13°C for 3 days, induced an antagonistic effect on microorganisms responsible for wine spoilage, like *Zygosaccharomyces*, *Lactobacillus kunkeei*, *H. uvarum*, and acetic acid bacteria (Chacon-Rodriguez et al., 2020). The work of Windholtz et al. (2021a) confirmed the implantation of the mixture of *M. pulcherrima* and *T. delbrueckii* on Merlot with its capacity to occupy the microbiological niche, leading to the decrease of fungal communities and the cultivable *H. uvarum* population. Chemical analysis of the wines obtained from Pinot noir underlined that bio-protection had no influence on the level of phenolic compounds and the volatile content of wines, suggesting that the bio-protection of must could also protect must and wines from oxidation (Simonin et al., 2020). Concerning the first available data on the application of bio-protection in the production of Rosé wines (grape variety Pinot noir), the results underlined the predominance of the bioprotective strain *M. pulcherrima* after pressing (representing more than 70% of the total yeast population), despite the high concentration of indigenous yeasts in the must. However, only the combination of the bio-protection and the addition of an antioxidant such as oenological tannins made it possible to preserve the colour of the wines (Puyo et al., 2023).

In many cases, field trials have proven the ability of bio-protection to reduce the development of spoilage flora, with the same effectiveness as adding sulfites. However, a number of recommendations have been suggested to ensure the effectiveness of this strategy: the maintenance of low temperatures during the pre-fermentation phases (Simonin et al., 2022), and considering the level of indigenous populations in grape must (Windholtz et al., 2021b). Indeed, a high wild microorganisms concentration (above 10^5^ CFU/mL) linked to advanced grape maturity can compromise the implantation of the bioprotective strain.

These limitations highlight the importance of investigating the mechanisms linked to the antagonistic effects of *M. pulcherrima* on the indigenous flora initially present in the must, in order to adapt and improve protocols for the winemaker’s benefit.

## 3. What specific mechanisms of *Metschnikowia pulcherrima* are involved in bio-protection?

Oenological conditions influence complex microbial ecosystems including yeasts and bacteria. The study of interactions inside these ecosystems present on must and during fermentation has become essential. A deeper understanding of these interactions is crucial to ensure better control of bio-protection in winemaking.

**Figure 2:**
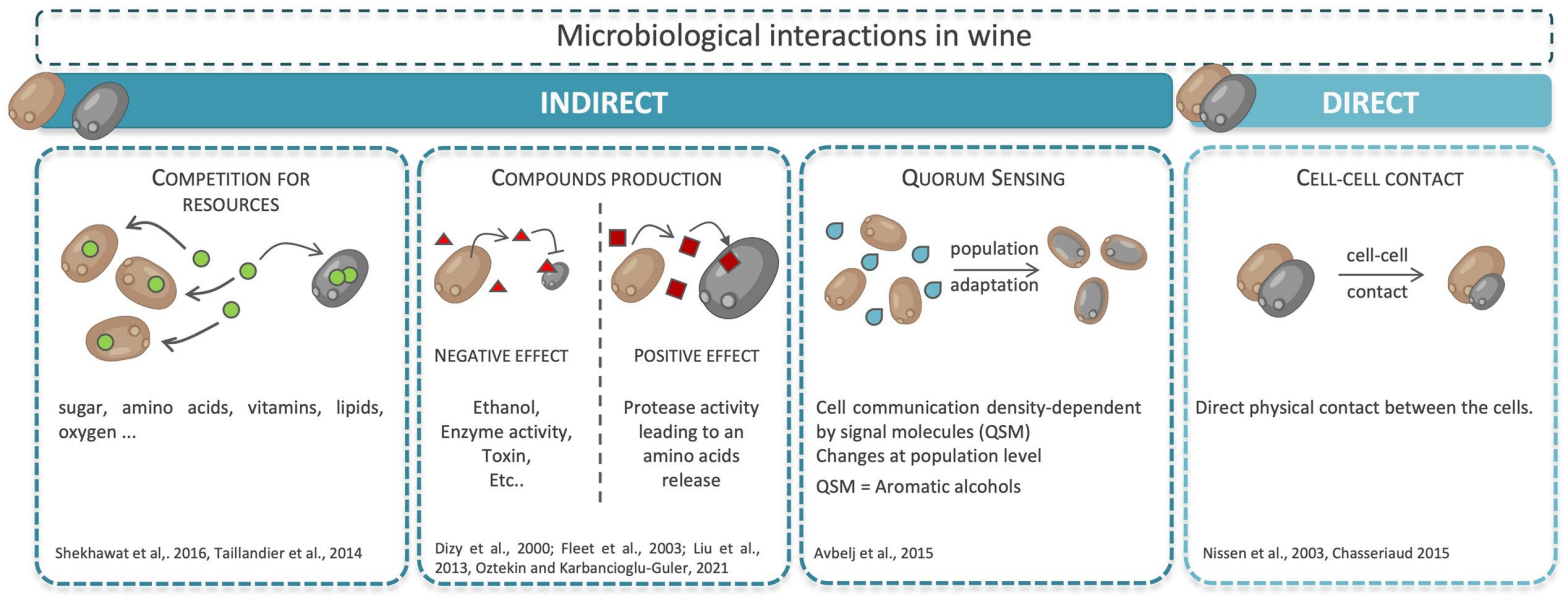
Microbial interactions in wine.

Interactions can be indirect, such as nutrient competition or the production of antimicrobial compounds, or direct interactions like cell–cell contact (Bordet et al., 2020; Zilelidou and Nisiotou, 2021) (Figure 2). Interactions are referred to as positive, neutral or negative. All populations involved can benefit from the interaction, or on the contrary be negatively affected. The effect of the interaction can also be asymmetric: one population can benefit from the interaction whilst the other population is negatively or not impacted. The effects of interaction impact the populations at different levels. Their growth parameters can be modified (latency phase, μmax value, maximal population, etc.), as can their metabolism (Sadoudi et al., 2017; Bordet et al., 2023).

Only few studies have focused on the investigation of direct interaction “cell–cell contact” in winemaking context (Kemsawasd et al., 2015a; Petitgonnet et al., 2019; Hu et al., 2022), and there is no evidence of such an interaction within the *Metschnikowia* genus. The only information suggesting a possible cell–cell contact interaction in *Metschnikowia* was given by Oztekin and Karbancioglu-Guler (2021) who showed the ability of some *M. pulcherrima* and *M. fructicola* strains to produce biofilm on polystyrene. Today, to explain the bio-protective action of NS yeasts in oenology, research focuses mainly on the potential mechanisms of indirect interaction.

### 3.1. Killer toxins

The production of antimicrobial peptides or proteins, such as killer toxins, was first reported in *Saccharomyces cerevisiae* (Bevan and Makover, 1963), and has been widely characterised since. Three main killer toxins have been reported in *S. cerevisiae*: K1, K2, and K28 (Novotna et al., 2004; Orentaite et al., 2016). Yeast killer toxin producers exhibit antimicrobial properties against other *S. cerevisiae* sensitive strains and are immune to their own killer toxin family (K1, K2, or K28), but could be sensitive to another killer family (Orentaite et al., 2016).

A killer phenotype has been reported in NS yeasts found in grape must and other environments (Santos and Marquina, 2004; Liu et al., 2012) (Table 2). Amongst these yeasts, some of them were reported to be killer toxin producers with antimicrobial activity on the main wine spoilage yeasts, *Brettanomyces* and *Hanseniaspora* genera (Mannazzu et al., 2019).

**Table 2:** Non-*Saccharomyces* yeast producing killer toxin.

Yeast	Toxin	References
*Kluyveromyces wickerhamii*	Kwkt	Comitini et al. (2004a, 2011)
*Wickerhamomyces anomalus* (formerly *Pichia anomala*)	Pikt and KW	Comitini et al. (2004a), De Ingeniis et al. (2009), Fernández de Ullivarri et al. (2014), and Abu-Mejdad et al. (2020)
*Candida pyralidae*	Cpkt1 and Cpkt2	Mehlomakulu et al. (2014, 2017)
*Tetrapisispora phaffi* (formerly *Kluyveromyces phaffi*)	Kpkt	Ciani and Fatichenti (2001) and Comitini et al. (2004b)
*Pichia membranifaciens*	Pmkt1 and Pmkt2	Belda et al. (2017)
*Torulaspora delbrueckii*	Tdkt and TK	Villalba et al. (2016) and Abu-Mejdad et al. (2020)
*Metschnikowia pulcherrima*	-	Büyüksırıt Bedir and Kuleaşan (2021) and Büyüksırıt-Bedir and Kuleaşan (2022)

Recently, the production of a killer toxin by a *M. pulcherrima* strain (TB26) isolated from grape vine was demonstrated (Büyüksırıt-Bedir and Kuleaşan, 2022). The application of the purified killer toxin was tested in ready to cook meatballs in order to extended their shelf life (Büyüksırıt Bedir and Kuleaşan, 2021). The same authors purified, characterised and tested different growth conditions for killer activity (Büyüksırıt-Bedir and Kuleaşan, 2022). Incubation at 20°C at a pH value of 7 showed the highest inhibition diameter on agar plates. An analysis by MALDI-TOF mass spectrometry gave a molecular weight of 10.3 kDa and provided the amino acid sequence of this toxin. The sequence comparison underlined that part of the sequence obtained (amino acids 31 to 50) showed a 100% correspondence score with the KHR killer toxin of *S. cerevisiae* characterised by Goto et al. (1990). Molecular weights of killer toxins are extremely variable. The size range reported in the literature can vary from 1.8 to 300 kDa (Kagiyama et al., 1988). The *M. pulcherrima* TB26 killer toxin seems to be in the low molecular weight range of the killer toxins characterised until now. These results were supported by Hicks et al. (2021) who tested the killer phenotypes of 11 *M. pulcherrima* strains. The authors showed that some strains exhibit a killer activity against *Escherichia coli*, *Salmonella enterica*, and *Staphylococcus aureus* on agar, an effect also achieved by adding the culture supernatant. They also analysed the protein extract of *Metschnikowia* culture and found a considerable amount of proteins with a size around 10 kDa, which corresponds to the killer toxin size identified by Büyüksırıt-Bedir and Kuleaşan (2022).

This approach remains to be explored with more strains of *M. pulcherrima* used for bio-protection in oenology. Farris et al. (1991) and Lopes and Sangorrín (2010) shed light on the killer phenotype (i.e., antagonist effect on agar plate by visualising a halo of inhibition) of *M. pulcherrima* strains isolated from grape, must, and the wine matrix. However, the killer phenomenon observed in those studies were not proved to be linked to the production of a peptidic killer toxin.

### 3.2. Pulcherriminic acid regulation and production

Microorganisms can produce non-peptidic compounds with antimicrobial properties. In the particular case of *M. pulcherrima*, the non-peptidic antimicrobial compound composed of four genes: *PUL1, PUL2, PUL3* is pulcherriminic acid. The first reports in the literature on pulcherriminic acid production date from the last century and concern yeasts and *Bacillus* bacteria (Kluyver et al., 1953; MacDonald, 1965; Uffen and Canale-Parola, 1972). The metabolic pathway involved in pulcherriminic acid synthesis has been well characterised since, especially in *Bacillus subtilis* and *Bacillus licheniformis*. Pulcherriminic acid is produced from two leucyl-tRNA that are cyclised by cyclodileucine synthase, encoded by the *yvmC* gene, which leads to the production of cyclo-(L-Leu-L-Leu). The latter is then oxidised in pulcherriminic acids by pulcherriminic acid synthase, encoded by the *cypX* gene (coding for a P450 cytochrome family protein). After its production in the cell, pulcherriminic acid is excreted into the extracellular medium by transporters encoded by the *yvmA* gene, where it can chelate iron ion (Fe^3+^) by a non-enzymatic reaction to form the red pigment named pulcherrimin (Wang et al., 2018; Yuan et al., 2020). The production of pulcherriminic acid is down-regulated by three main genes in *Bacillus*: *yvnA*, *yvmB* and *abrB*. Moreover, Wang et al. (2018) showed that the iron concentration of the medium impacts pulcherriminic acid production. These authors have shown that an iron-limited medium inhibited the *yvmC-cypX* cluster and thus inhibited pulcherriminic acid production.

**Figure 3:**
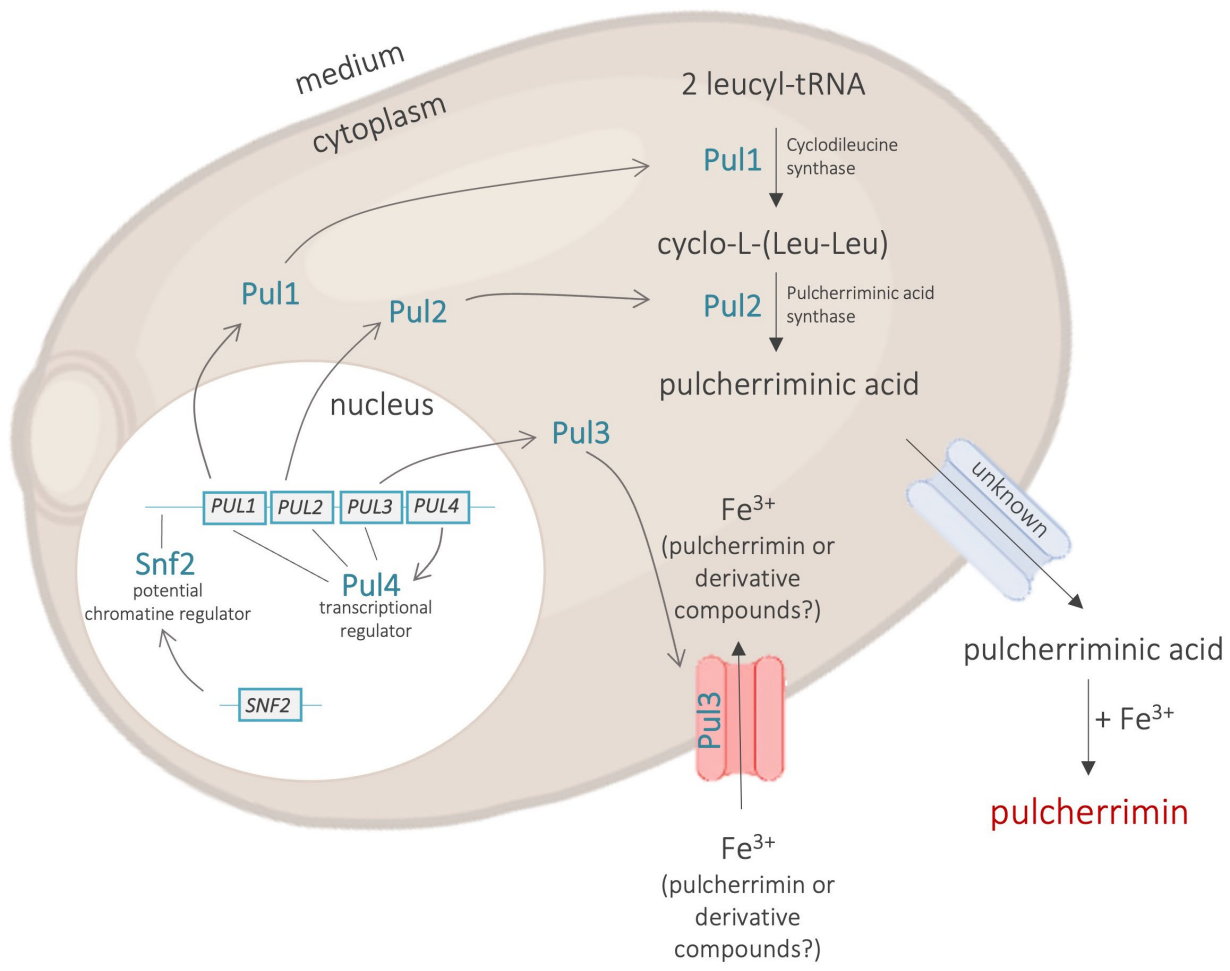
Pulcherriminic acid production by *Metschnikowia pulcherrima.*

In *M. pulcherrima*, as well as in other pulcherrimin-producer *Metschnikowia* species, the metabolic pathway of pulcherriminic acid production is not yet as well-known as in *Bacillus.* Having prior knowledge of the genes involved in the synthesis of this pigment in *Bacillus*, Piombo et al. (2018) searched for homologous genes by sequence alignment in two newly sequenced *M. fructicola* strains. But the results were negative. Genes that may be involved in the production of this antimicrobial pigment in *Metschnikowia* were identified by Sipiczki (2020). The *PUL* (for PULcherrimin) gene cluster was identified as being involved in the production of pulcherriminic acid in *Metschnikowia*, *Kluyveromyces*, and *Zygotorulaspora* genera (Krause et al., 2018). This cluster is supposedly composed of four genes: *PUL1*, *PUL2*, *PUL3* and *PUL4* (Figure 3). Krause et al. (2018) were able to assign potential functions to each of these genes. The *PUL1* gene is found to be potentially responsible for the production of cyclo-(L-Leu-L-Leu) from two leucyl-tRNA (corresponding to *yvnB* in *Bacillus*), followed by *PUL2* acting to oxidise the cyclodipeptide into pulcherriminic acid (corresponding to *cpyX* in *Bacillus*). The *PUL4* gene encodes a transcriptional regulator of *PUL1, PUL2* and *PUL3* genes. The *PUL3* gene appears to be involved in the transport of iron in pulcherrimin, from outside to inside the cell for its reuse in cell metabolism. Studies conducted on *Bacillus* seem to indicate that iron once chelated by pulcherriminic acid becomes unavailable for the other microorganisms as well as for the bacteria producing pulcherrimin, unlike for yeasts producing this pigment which seem to possess a mechanism allowing them to reuse the iron in the medium even after complexation with pulcherriminic acid. The work of Krause et al. (2018) also showed the presence of genes homologous to *PUL3* and *PUL4* in other genera and species, such as *S. cerevisiae*, *Lachancea thermotolerans,* and *M. bicuspidata*. Some of them have both genes, and others have only *PUL4*. Species with only *PUL4* are not able to reuse iron once chelated by pulcherriminic acid, unlike strains that have both genes (*PUL3* and *PUL4*). Amongst them we find *S. cerevisiae* which has *PUL3* and *PUL4* and seems to be able to reuse iron despite the presence of pulcherriminic acid in the medium. It is hypothesised that *PUL3* encodes the transporter that allows the internalisation of pulcherrimin and that *PUL4* regulates the transcription of *PUL3* by binding DNA (Krause et al., 2018).

The genes of the *PUL* cluster do not appear to be the only ones involved in the production of pulcherriminic acid. Using the PacBio method, Gore-Lloyd et al. (2019) sequenced a new strain of *Metschnikowia*, identified as a *Metschnikowia* aff. *pulcherrima* strain, as well as three spontaneous pigmentless mutants of this strain. Genome comparison between the wild-type strain and the pigmentless mutants showed a single mutation in the gene homologous to the *SNF2* gene in *S. cerevisiae*. This mutation introduces a premature stop codon in the sequence, leading to the production of a truncated and therefore non-functional protein. In *S. cerevisiae*, *SNF2* codes for a transcriptional regulator by chromatin remodelling (Snf2) (Hirschhorn et al., 1992). This mutation seems to impact the transcription of many genes including genes of the *PUL* cluster. Indeed, strains mutated on the *SNF2* gene show a strong decrease of *PUL* cluster gene transcription. Since Snf2 is involved in chromatin remodelling, it is possible that the non-functional Snf2 prevents the remodelling of chromatin at the *PUL* cluster loci, and thus decreases the transcription of these genes (Gore-Lloyd et al., 2019).

Iron chelation by pulcherriminic acid makes this resource unavailable for microorganisms in the environment, and thus negatively impacts the growth of microorganisms needing Fe^3+^. The secretion of pulcherriminic acid into the medium by pulcherrimin producing yeasts, such as *M. pulcherrima*, could partially explain the antagonistic effect of this species on some microorganisms. The antagonistic effect of pulcherrimin was therefore studied with different species of yeasts producing this pigment. These studies were conducted *in vitro* (mostly with agar plates) and *in vivo*. Different species of *Metschnikowia* (*citrensis*, *fructicola*, *pulcherrima*) have shown an inhibitory effect on microorganisms that can alter the fruit or the resulting food products (Türkel and Ener, 2009; Hicks et al., 2021; Wang et al., 2021; Zhang et al., 2023). It was shown that the intensity of the pigmented halo around *Metschnikowia* on agar increases with the iron concentration in the medium, contrary to the inhibition zone which decreases when increasing the iron concentration. Indeed, in a medium with excess iron, Fe^3+^ chelation by pulcherriminic acid will not be sufficient to cause a deficiency in the environment, and thus not be deleterious for the surrounding microorganisms (Sipiczki, 2006; Saravanakumar et al., 2008; Türkel and Ener, 2009; Wang et al., 2021). Furthermore, Kregiel et al. (2022) showed that pulcherrimin has no antimicrobial effect. Their results support the hypothesis that it is iron chelation and not the pigment itself that has an inhibitory effect. In the oenological context, Oro et al. (2014) showed that the production of pulcherriminic acid by *M. pulcherrima* had an inhibitory effect on non-*Saccharomyces* yeasts found in oenology, including spoilage yeasts such as apiculate yeasts (*Hanseniaspora guillermondii*) and *Brettanomyces bruxellensis*.

Under the conditions tested, the production of pulcherriminic acid seems to explain most of the antagonistic effect of *Metschnikowia* observed. However, the production of this compound does not seem to be the only explanation for its antimicrobial effect. Indeed, in studies that sought to confirm the involvement of iron chelation in inhibitory effects, the authors supplemented the medium with iron or induced the knock-out or down-regulation of genes involved in pulcherriminic acid production. In most cases, the inhibitory effect is strongly reduced (by 80%) (Gore-Lloyd et al., 2019; Wang et al., 2021), but not always completely eliminated (Gore-Lloyd et al., 2019). This suggests that iron chelation is not the only negative interaction that *M. pulcherrima* establishes in playing its bioprotective role.

### 3.3. Quorum sensing

Quorum Sensing (QS) is intercellular communication mediated by the excretion of a density-dependent signal molecule. Once the target cell density is reached, the signal molecule concentration also reaches a threshold value, inducing signal transduction pathways that coordinate a response at the population level rather than at the cell level. This communication mechanism was first discovered in *Vibrio fischeri* for bioluminescence production (Nealson et al., 1970; Dunlap, 1999). In bacteria, QS has been found to be involved in different cellular mechanisms such as biofilm formation and the production of enzymes and other compounds required for pathogenicity or growth regulation (Miller and Bassler, 2001).

Bacteria are not the only microorganisms able to regulate their gene expression at the population scale to adapt to their environment. QS is also an interaction phenomenon investigated in yeasts, and more extensively in the pathogenic yeast *Candida albicans*, as it controls various cellular transformations such as biofilms, transitions between cellular growth and stationary phases, hyphal production, and many others (Kügler et al., 2000; Chen and Fink, 2006; Hogan, 2006; Sprague and Winans, 2006). In yeasts, it seems that QS is induced by aromatic alcohols in response to environmental factors such as a low nitrogen level in the medium. In the model yeast *S. cerevisiae*, the Quorum Sensing Molecules (QSMs) found are 2-phenylethanol and tryptophol. Tyrosol has been found in *C. albicans* but its role as a QSM in *S. cerevisiae* is still controversial (Chen and Fink, 2006; Wuster and Babu, 2009; Zupan et al., 2013; Avbelj et al., 2016; Padder et al., 2018; Mehmood et al., 2019; Jagtap et al., 2020). These molecules are produced from phenylalanine, tryptophan and tyrosine respectively, through the Ehrlich pathways. In order to produce these QSMs, amino acids must undergo three phases of transformation: transamination, decarboxylation and oxidation, involving the genes *ARO8*/*ARO9*, *ADH*, and *ARO10,* respectively (Sprague and Winans, 2006; Hazelwood et al., 2008; Wuster and Babu, 2009; Avbelj et al., 2015; Jagtap et al., 2020). *Saccharomyces cerevisiae* and *C. albicans* are not alone in being assumed to be capable of communicating through QS. Pu et al. (2014) showed that the bioprotective effect of *H. uvarum* on lemon through biofilm formation in fruit wounds was induced by phenylethanol secretion and thus under the potential control of QS. Furthermore, other yeasts and fungi are suspected of being able to use QS for their intercellular communication, mainly through aromatic alcohol production (Kügler et al., 2000; Sprague and Winans, 2006; Gori et al., 2011; Padder et al., 2018).

Except for *C. albicans*, this type of interaction remains less studied and understood in yeasts. The existence of QS as well as the role of aromatic alcohols as signalling molecules remains controversial and requires further research (Winters et al., 2019, 2022). Nevertheless, it has been shown that some non-*Saccharomyces* wine yeasts, including *M. pulcherrima*, are able to produce aromatic alcohols suspected of playing the role of QSMs in *S. cerevisiae* (González et al., 2018; Petitgonnet et al., 2019). It could therefore be interesting to extend research in *M. pulcherrima* to the involvement of these molecules as potential QSMs and to their control of gene expression amongst non-*Saccharomyces* yeasts mainly present on a grape must.

### 3.4. Enzymatic activities in *Metschnikowia pulcherrima*: a potential role in bio-protection?

Amongst the NS yeasts, *Metschnikowia pulcherrima* stands out due to its substantial production of a very diverse range of enzymatic activities, such as amylase, lichenase, cellulase, lipase, and glucanase (Charoenchai et al., 1997; Strauss et al., 2001; Oztekin and Karbancioglu-Guler, 2021). Saravanakumar et al. (2008) showed that a *M. pulcherrima* strain isolated from an apple carposphere is able to secrete chitinase in the medium. This enzymatic production was found to be involved in its biocontrol activity on *Botrytis cinerea*. Other studies have also reported chitinase (Banani et al., 2015; Pretscher et al., 2018; Freimoser et al., 2019; Morata et al., 2019) and β-1,3-glucanase productions (Oztekin and Karbancioglu-Guler, 2021) by yeasts belonging to the *Metschnikowia* genus, justifying their potential use as biocontrol agents on vegetables and fruits, as well as grapevine. To our knowledge, enzyme production by *M. pulcherrima* and potential enzymatic activity levels have never been tested in relation to the acidity associated with oenological conditions, in order to verify the possible implication of extracellular enzymes synthesised by the yeast in a bio-protection context.

### 3.5. Oxygen needs and possible competition

Oxygen plays a key role in the metabolism of NS yeasts. At the pre-fermentative stage, the level of dissolved oxygen (DO) in must is about 8 mg/L (at 20°C) and decreases during alcoholic fermentation. Depending on oenological practices, the concentration of DO added to the must can vary and practices such as punching down and pumping over to the vat incorporate significative quantities of oxygen into the medium (Moenne et al., 2014).

Many NS yeasts are high oxygen consumers (Visser et al., 1990). Quirós et al. (2014) studied the respiratory quotient (RQ) of *S. cerevisiae* and many NS yeasts. Amongst the yeasts studied, they showed that at the beginning of alcoholic fermentation when oxygen is available in the must, *M. pulcherrima* has an RQ of 1. This RQ means that all the sugar metabolised by the yeast is respired and not fermented, in contrast to *S. cerevisiae* which has an RQ of 4 (meaning that only about 10% of the sugar consumed is respired). The authors also showed that the RQ of *M. pulcherrima* could reach a maximum fermentative capacity of 2.6, which indicates that the yeast may consume at least 17% of the sugar by respiration.

Several studies have investigated the role of oxygen in the persistence and survival of yeasts in co-cultures with *S. cerevisiae*. Most of the time, NS yeasts do not persist in must, especially after inoculation with a *S. cerevisiae* strain. In the literature, the addition of oxygen has been found to improve the persistence of NS yeasts in the medium (Holm Hansen et al., 2001; Shekhawat et al., 2016, 2018; Englezos et al., 2018; Yan et al., 2020). Morales et al. (2015) and Shekhawat et al. (2016) showed that the decline of *M. pulcherrima* in co-culture with *S. cerevisiae* is delayed by increasing O_2_ supply in white grape juice and synthetic must. Although these studies focused on oxygen requirements during co-cultures combining *S. cerevisiae*/non-*Saccharomyces* yeast, they nonetheless highlighted the importance of oxygen needs for NS yeasts. In order to better understand and control the bioprotective effect of *M. pulcherrima*, it appears essential to quantify its oxygen requirement, as well as those of the indigenous flora, such as *Hanseniaspora* yeasts, with the aim of optimising the implantation of the bioprotective strain in relation to its efficiency. High oxygen consumption exhibited by *M. pulcherrima* yeasts could induce competition between bioprotective and indigenous yeasts, leading to the inhibition of potential spoilage yeasts.

### 3.6. Nutrient requirement: a path to competition

Oxygen is not the only resource consumed by yeast, which could lead to competitive phenomena. Amongst these nutrients, requirements for nitrogenous resources (ammonium and amino acids) are those studied most, playing a central role in yeast metabolism synthesising protein and nitrogenous bases, and in the production of aromatic compounds by the Ehrlich pathway (Hazelwood et al., 2008). As for oxygen, competition for nitrogen compounds could be involved in the negative interactions triggered by the bioprotective yeast.

Nitrogen sources are classified as “preferential” and “non-preferential” resources. Preferential resources are consumed in priority by the yeasts and support their growth more efficiently, contrary to non-preferential resources which are consumed once the preferred resources have been entirely consumed or have become limited in quantity in the medium (Crépin et al., 2012). The regulation of nitrogen consumption has been extensively studied in *S. cerevisiae* and appears to be regulated by two main systems: the Nitrogen Catabolic System (NCR) and regulation involving the Ssy1-Ptr3-Ssy5 (SPS) sensor of the plasma membrane (Crépin et al., 2012).

In NS yeasts, the regulatory pathways involved remain poorly investigated. Moreover, nitrogen requirements were found to be highly variable between yeast species but also strain-dependent (Kemsawasd et al., 2015a; Gobert et al., 2017; Prior et al., 2019; Roca-Mesa et al., 2020; Seguinot et al., 2020). The data heterogeneity can be explained by the great diversity of experimental conditions: variable temperatures, different strains analysed as well as different growth media (grape juice, synthetic must or synthetic medium). According to Gobert et al. (2017), the preferential sources of *M. pulcherrima* in grape juice at 28°C are Ile, Leu, Lys, Met, Glu and Cys, whilst at 20°C the preferential resources are Ala, Cys, Glu, His, Lys and Thr. In the synthetic medium YNBMAF at 25°C, the resources consumed are mainly Ala and Asn (Kemsawasd et al., 2015b). In synthetic must at 22°C Lys is found mainly consumed with Glu, Gln, His and Val in medium with amino acids and ammonium, or with Phe in medium with only amino acids as nitrogen sources (Roca-Mesa et al., 2020). Furthermore, Seguinot et al. (2020) showed that consumption also varies according to the initial nitrogen concentration, with an increase of the consumption of resources with the concentration of nitrogen in the environment.

To the best of our knowledge, there are still no studies that have attempted to demonstrate competition for nitrogen resources in a bio-protection strategy. Better understanding of the nitrogen requirements of *M. pulcherrima* in relation with the environmental conditions of growth is essential in order to investigate whether potential competition for nitrogen compounds may be involved in the negative effect on the growth of indigenous yeasts.

In must, other compounds could lead to competition, such as lipids or vitamins. Lipids are necessary for the maintenance of cell membrane integrity and they improve resistance to the stresses induced by ethanol. Moreover a medium limited in lipids can lead to languishing fermentation (Tesnière, 2019; Mbuyane et al., 2022). Vitamins are also an important factor for yeast growth, and a medium limited in vitamins could also lead to sluggish fermentation. Also, vitamin consumption appears to be species- and strain-dependent (Evers et al., 2021). The lipid and vitamin requirements of NS yeasts are still poorly explored and deserve further investigation to determine if they are involved in the bioprotective phenomenon.

## 4. Conclusion and perspectives

Non-*Saccharomyces* yeasts are found to be predominant on grapes and must during the early stages of fermentation, before being replaced by *S. cerevisiae* which will complete alcoholic fermentation. NS yeasts have long been criticised for their negative effects on the organoleptic qualities of wine, but numerous studies have subsequently highlighted many properties of considerable oenological interest amongst NS yeasts. *Metschnikowia pulcherrima* presents numerous advantages for the wine industry and is now distinguished by its effectiveness in the bio-protection of musts, making it a possible alternative to the use of sulphites to protect against indigenous yeasts, potential spoilage agents.

Although much research has highlighted the physiological characteristics of this yeast that may play an anti-microbial role, it remains difficult to predict and ensure perfect efficiency under cellar conditions. Indeed, Windholtz et al. (2021b) showed that on must from grapes with advanced maturity, bio-protection was not sufficient to limit the proliferation of indigenous flora. It is important to study in greater detail the resource requirements of bioprotective yeasts as well as those of indigenous flora to better understand the mechanisms and the limits of bio-protection. But many other interactions can lead to the inhibition of spoilage microorganisms in the must. Indeed, many questions remain concerning the production of toxic compounds by *M. pulcherrima*, such as potential killer toxins, and the existence of quorum sensing in this yeast. In addition, other non-*Saccharomyces* yeasts have shown the ability to make cell–cell contact interactions. *Metschnikowia pulcherrima* was found to be able to adhere to a polystyrene surface (Parafati et al., 2015) which could suggest its capacity to induce adhesion between cells and surfaces. Cell–cell interaction has never been demonstrated in the context of bio-protection with *M. pulcherrima*, but given its surface adhesion properties, this appears to be an important topic to investigate. The genetic diversity of *M. pulcherrima* is still little known and a recent study tended to indicate that the genus *Metschnikowia* deserve more detailed study to better understand strain diversity and distribution within the different species of this genus (Sipiczki, 2020).

In addition, the perfect protection of a grape must also includes protection against oxidation. As reported in this review, *M. pulcherrima* is described in the literature to be a strong consumer of oxygen. The high consumption of this resource could make O_2_ rapidly unavailable in the grape must and thus prevents oxygen from entering the redox pathways that leads to wine browning and to the production of undesirable aromas. In addition, *M. pulcherrima* is known to secrete pulcherriminic acid, which once in the medium chelates Fe^3+^. This ion is also involved in redox mechanisms through the Fenton reaction. The depletion of iron in the medium by pulcherriminic acid could also contribute, to a lesser degree, to protecting the matrix against oxidation. The literature has also highlighted interaction phenomena between the yeast cell wall and anthocyanins. These interactions are species-dependent and impact the final colour of the wine (Morata et al., 2019; Vicente et al., 2020). More information on the effect of *M. pulcherrima* on must and wine colour, and on its combination with other antioxidant compounds, could also be crucial for professionals to optimise its application in the wine sector.

## Author contributions

All authors listed have made a substantial, direct, and intellectual contribution to the work and approved it for publication.

## Conflict of interest

The authors declare that the research was conducted in the absence of any commercial or financial relationships that could be construed as a potential conflict of interest.

## Publisher’s note

All claims expressed in this article are solely those of the authors and do not necessarily represent those of their affiliated organizations, or those of the publisher, the editors and the reviewers. Any product that may be evaluated in this article, or claim that may be made by its manufacturer, is not guaranteed or endorsed by the publisher.
